# The relationship between transformational leadership, work motivation, and engagement among nurses in Jordanian governmental hospitals

**DOI:** 10.1186/s12912-025-03518-7

**Published:** 2025-07-04

**Authors:** Roa’a Abu-Qutaish, Mohammad R. Alosta, Ghada Abu-Shosha, Islam Ali Oweidat, Abdulqadir J. Nashwan

**Affiliations:** 1 Faculty of Nursing, Zarqa University, Zarqa, Jordan; 2 Nursing & Midwifery Research Department (NMRD), Hamad Medical Corporation, Doha, Qatar; 3 Department of Public Health, College of Health Sciences, QU Health, Qatar University, Doha, Qatar

**Keywords:** Transformational leadership, Nurses, Work motivation, Work engagement, Jordan

## Abstract

**Introduction:**

The healthcare sector faces persistent challenges related to workforce management, including maintaining high levels of motivation and engagement among nurses. Transformational leadership is one of the primary leadership styles that has emerged as a key factor affecting nurses’ motivation and work engagement. However, there is a lack of evidence on the relationship between these variables, particularly in Jordan.

**Aim:**

The current study examines the relationship between transformational leadership, work motivation, and work engagement among nurses working in governmental hospitals in Jordan.

**Method:**

To answer the research questions, a descriptive, cross-sectional, correlational design was used. A convenience sampling technique was used to recruit nurses from three governmental hospitals. The Transformational Leadership scale, the motivation at work scale, and the work engagement scale were used to measure the study’s main variables.

**Result:**

A total of 125 nurses participated in the study. The study results showed that nurses exhibit moderate transformational leadership levels 36.80 ± 15.84, moderate motivation levels 51.57 ± 16.75, and moderate to high engagement levels 80 ± 22.65. The results also showed that there was a significant positive correlation between transformational leadership and work motivation (*r* = .49, *p* < .001) and between transformational leadership and work engagement (*r* = .41, *p* < .001). The study’s findings also indicated that a transformational leadership style, years of experience, and working hours per week significantly predicted nurses’ work motivation and engagement. Transformational leadership was positively associated, while years of experience and working hours were negatively associated with both outcomes. The models explained 31.1% of the variance in motivation and 34.2% of the variance in engagement (*p* < .001).

**Conclusion and implications:**

Transformational leaders foster a supportive and inspiring work environment that can significantly boost nurse motivation and engagement. This can improve healthcare quality and address nursing workforce challenges such as high turnover and staff shortages. The results suggest that healthcare administrators and policymakers should prioritize developing and implementing transformational leadership training programs for all nurse leaders as a strategic approach to enhance nurses’ motivation and engagement.

**Clinical trial number:**

Not applicable.

## Background

Effective leadership in nursing is crucial for cultivating a favorable work environment that fosters professional advancement and satisfaction, increasing nurses’ productivity and performance, facilitating care coordination, and ensuring the delivery of high-quality nursing care to meet patients’ health needs [1–3]. Since leadership is a key component of care management success, qualified professionals must prioritize work effectiveness, management process, and the supply of sufficient material and physical resources to ensure better healthcare outcomes [4].

The US Institute of Medicine (IOM) identified nurse leadership as key to achieving the national healthcare goal [5]. Krepia and Katsaragakis [6] have identified transformational leadership (TL) as one of the primary leadership styles with the potential to bring about change, improvement, and innovation in nursing practice. This style is characterized by utilizing motivation and moral principles to motivate followers to commit themselves to organizational objectives [7], resulting in empirically positive outcomes for patients, healthcare professionals, and organizations [8]. Moreover, transformational leaders incorporate nursing beliefs into shared moral standards, enabling them to understand and address their needs, which leads to positive outcomes such as increased job satisfaction and lower turnover rates [9].

Transformational leaders are categorized into four elements: idealized influence, in which a leader acts with trust and integrity; inspirational motivation, in which a leader motivates others by creating a shared vision; intellectual stimulation, in which a leader thinks outside the box and encourages others to look for other ideas; and individualized consideration, in which leaders treat others as individuals [10]. These elements collaborate to create an environment in which employees feel valued, motivated, and engaged, resulting in enhanced organizational outcomes.

Transformational leadership has become a key factor affecting nurses’ motivation and engagement. Carthon and Hatfield [11] found that staff nurses became motivated and engaged when they witnessed the actions of transformational leaders, prompting them to adapt their self-perceptions, beliefs, and values to align with those of their leaders. Baljoon et al. [12] defined motivation as the “force” behind nurses’ behavior, distinguishing between intrinsic motivation, where nurses engage in a behavior because they find it inherently rewarding, and extrinsic motivation, where nurses are driven to perform a behavior or activity to obtain a reward for work engagement or avoid punishment. Furthermore, the same study reported that several variables can affect the level of motivation demonstrated by nurses, such as age, years of experience, autonomy, educational level, and work positions, notwithstanding the extent to which they are empowered, remunerated, supervised, promoted, and supported by their leaders [12].

When motivated, nurses become more engaged and invested in the structure and processes underlying the healthcare organization’s performance. They view themselves as essential and valued components to operate such systems [13]. Work engagement is defined as a constructive and fulfilling cognitive state related to one’s job, characterized by three distinct aspects: absorption is marked by focus and deep engagement in work, vigor reflects the motivation to exert effort, and dedication is associated with participation, and the drive to invest energy in work relates to vigor [14]. In addition, work engagement is a concept that has been increasingly utilized in nursing to describe the sense of pride, commitment, and dedication of a nurse to one’s employer or organization [15]; essentially, being engaged meant nurses were involved beyond the scope of their job responsibilities and were empowered to support healthcare organizations [16].

The healthcare sector faces persistent challenges related to workforce management, particularly among nurses. As frontline workers, nurses are positioned to deliver direct care to patients, families, and other service users, yet they frequently experience and must adapt to substantial challenges [17]. These challenges include workload, staff shortages, high turnover intentions, retention difficulties, and inadequate or insufficient compensation [18]. Moreover, amidst the stresses of the healthcare service on the one hand and the demands of meeting workload on the other, nurses often face constraints that limit their ability to maintain a high quality of life and demonstrate motivation and engagement in their job [19].

Furthermore, leadership styles can significantly impact a variety of outcomes for nurses. A systematic review conducted by McCay and Lyles [5] found that leadership styles, such as transformational, transactional, situational, and authentic styles, can lead to diverse outcomes in nurse job satisfaction, staff motivation, work engagement, and the ability to provide safe patient care. Moreover, several reports have highlighted the importance of supportive work environments in influencing nurses’ outcome measures, indicating a need for effective leadership styles that improve the working environment for nurses [20]. The findings of Atalla et al. [21] further support this by showing how leadership that fosters teamwork, responsibility, workload management, and empowerment can reduce the incidence of missed nursing care, thus improving nurse and patient outcomes.

There is no evidence for the relationship between TL, nurse motivation, and nurse engagement. Instead, studies have explored the relationships between leadership and other substitute variables (e.g., nurse satisfaction, nurse turnover, intention to leave, job performance, etc.) that imply measures of motivation and engagement but without directly assessing them [22–24]. Moreover, no studies have globally investigated the relationships among all variables, and none have critically examined the directions, nature, extent, and characteristics of these relationships as they relate to nurse practices and direct patient care.

This lack of evidence creates a significant gap in the literature, potentially influencing our understanding of TL, nurse motivation, and nurse engagement in the Jordanian healthcare context. Furthermore, a lack of clarity regarding the relationship between TL, motivation, and engagement can create unsupportive and challenging environments, which may hinder nurses from fulfilling their roles and responsibilities. This, in turn, can result in compromised organizational processes, diminished staff morale and satisfaction, and adverse health outcomes for patients, families, and other service users [25–27].

Addressing the gap in the existing literature on TL and its relationship with work motivation and engagement significantly impacts healthcare organizations, nursing practice, and patient care. Unlike previous studies that only implied these relationships through substitute variables, this study directly investigates the association between transformational leadership, work motivation, and engagement. It is the first to do so within the context of Jordanian governmental hospitals, where high patient volumes and limited resources create a unique and pressing need for evidence-based leadership practices.

This study offers empirical evidence relevant for developing leadership strategies and policies tailored to the Jordanian healthcare system. Nursing leaders can leverage the findings to understand the importance of adopting TL in fostering a positive work environment, which can enhance staff motivation and engagement, ultimately leading to improved healthcare quality, decreased turnover rates, and alleviating staff shortages in the nursing profession [28].

Further, nurse educators can benefit from the study by using its results to conceptualize, develop, plan, and implement educational workshops and training sessions to enable nursing leaders to gain the confidence and competence to demonstrate TL that aligns with their personal attributes and organizational demands.

The study’s results can be utilized by nurse leaders, policymakers, and hospital executives to institutionalize the need for TL that addresses work conditions and meets the needs of motivating and engaging nurses to improve the quality of healthcare services. While staff nurses can initiate engaging with activities and learning opportunities that will enhance their capabilities to lead, sustaining such engagement would not be possible without managerial and executive support. Finally, the study’s findings can benefit patients, their families, and other service users as staff nurses advocate for safe and high-quality practices, protecting patients from potential threats that could impact their hospital stay, recovery, and overall health outcomes. Therefore, the current study examines the relationship between nurses’ perceived level of TL, work motivation, and work engagement among nurses working in governmental hospitals in Jordan.

## Research questions

The study aims to answer the following questions:

**RQ 1**. What is the level of nurses’ perception toward transformational leadership in governmental hospitals in Jordan?

**RQ 2**. What are the work motivation and engagement levels among nurses working in governmental hospitals in Jordan?

**RQ 3**. Is there a relationship between nurses’ perceived level of transformational leadership, work motivation, and work engagement among nurses working in governmental hospitals in Jordan?

**RQ 4**. What are the predictors of work motivation and engagement among nurses working in governmental hospitals in Jordan?

## Methods

### Research design

A descriptive, cross-sectional, correlational design was used to address the research questions.

### The study setting, population, sampling, and sample size

The study was conducted in three governmental hospitals; the target population was all nurses working there during the research period. These hospitals are located in the central region and were selected based on the number of beds (≥ 350 beds) [29]. A convenience sampling technique was used to recruit participants due to its practicality and feasibility in accessing eligible nurses within the available research timeframe. The sample comprises those who agree to participate in the research from the accessible population. The inclusion criteria are as follows: being a registered nurse, working at any ‘hospital’ wards or units, and having a minimum of one year experience in the same hospital to ensure that participants are familiar with the hospital protocols; regarding the exclusion criteria: any participant who occupies a managerial, educational, or executive position.

To determine the minimum sample size of the study participants, the researcher calculated it based on a multiple regression analysis with a conventional power of 0.80, a conventional criterion of statistical significance (α) of 0.05, and a medium effect size of 0.15, using G*Power 3.1.9.7 [30] with nine predictors. The sample size in this study was initially estimated to be 114. An additional 10% of the participants were added to overcome the incomplete questionnaire, resulting in a sample size of 125.

### Measurement tools

Measurement tools that were carefully chosen from the literature and met the variables used in this investigation were utilized to collect data for this study. The questionnaire consists of four parts, including a participant demographic sheet, the Transformational Leadership Questionnaire, the Motivation at Work Scale (MAWS), and the Utrecht Work Engagement Scale (UWES).

#### Participant demographic sheet

The participant demographic sheet was utilized to collect data on the participants’ sociodemographic characteristics, namely age, gender, educational attainment, working department, years of experience as a registered nurse, years of experience in the current department, salary, and working hours per week. In particular, documenting the distribution of nurses across different hospital units provides essential context for interpreting potential variations in perceptions of transformational leadership, work motivation, and engagement across diverse clinical environments.

#### Transformational leadership questionnaire

The instrument used to measure nurses’ perceived level of TL was adapted from the Multifactor Leadership Questionnaire [31] utilizing 20 items that measure four items: the idealized influence dimension (6 items), the inspirational motivation dimension (5 items), the intellectual stimulation dimension (5 items) and individualized consideration dimension (4 items). The scale is based on a 5-point Likert-type response pattern in which the response categories range from (0) not at all to [4] frequently, if not constantly. Besides, no cut-off scores were set by the original tool developers. However, higher scores indicated more significant use of TL style within the respective units or departments. Bass and Avolio [31] reported that reliability for the components ranged from 0.73 to 0.94, and in the current study, it was found to be 0.97.

#### Motivation at work scale (MAWS)

The instrument developed by Gagné, Forest [32], the MAWS, consists of 12 items and has four subscales. Each subscale consists of three items, i.e., intrinsic motivation (items 4, 8, & 12), identified regulation of extrinsic motivation (items 3, 7, & 11), introjected regulation of extrinsic motivation (item 2, 6, & 10), and external regulation of extrinsic motivation (item 1, 5, & 9). The rating scale ranges from 1 (not at all) to 7 (exactly). The mean score results can be categorized as follows: low motivation level (1-3.58), moderate motivation level (3.59–4.43), and high motivation level (4.44-7). This tool has been used in different studies, and the total scale reliability coefficient ranged from 0.81 to 0.89 [32,33], and in the current study, it was found to be 0.96.

#### Utrecht work engagement scale (UWES)

This tool was developed by Schaufeli and Bakker [34] to measure work engagement. It consists of 17 items, including three defined subscales: Vigor (six items), which represents energy levels at the time of working, sustained effort at work, and persistence; Dedication (five items), which shows the relevance of work to a worker’s life as well as pride, inspiration, and enthusiasm toward what nurses do, Absorption (six items) that shows focus on performed activities work satisfaction and compliance with responsibilities. A 7-point Likert scale, ranging from 0 (Never) to 6 (Always), is used to measure nurses’ perception regarding work engagement. The total score ranges from 0 to 102; the higher the score, the more nurses are engaged in their work [34]. According to the reliability [35], reported a reliability coefficient of 0.84 for the total scale, with subscale reliabilities ranging from 0.76 to 0.89. In the current study, it was found to be 0.96.

### Data collection procedure

Data collection commenced after obtaining ethical approval. Then, the researcher contacted the nursing director in each hospital to explain the purpose, method, and data collection procedures. Once permission was obtained, the researcher coordinated with the heads of departments where the study was carried out. A master list of eligible nurses based on the inclusion-exclusion criteria was obtained. The data were collected between May and June 2024 via an online self-reported questionnaire designed via Google Forms, and all items were marked as obligatory. The link to the data collection kit, including the cover letter and questionnaire, was distributed to all selected departments by the nurse manager either via email or through the designated WhatsApp group. The expected time needed to complete the questionnaire is 10–15 min.

### Ethical considerations

The Institutional Review Boards (IRB) of Zarqa University’s/ Faculty of Nursing (12/2023) approved this project. This ensures that the study strictly adheres to ethical guidelines and criteria for conducting research. Nurses have the autonomy to join the study, and participation is voluntary. Those nurses who initially agreed to participate can withdraw from the study at any time without negatively affecting their careers. The author treated responding to the electronic link as consent to participate, collected no personally identifiable information, and only a numerical code identified the participant. In addition, the researcher stored all electronic responses on an encrypted and password-protected laptop computer, keeping them private and confidential. Finally, permissions from the original authors to use the questionnaires in the study were obtained.

### Data analysis

After collecting nurses’ questionnaires, SPSS version 26 was used to analyze the data. The author conducted a full review, including data screening, cleaning, coding for categorical data, and managing the outlier values. After that, the data were assessed for normality and the underlying assumptions required for parametric statistical tests; the results indicated that the data met the normality assumption for most variables. Descriptive statistics were used to measure sociodemographic characteristics’ frequency, mean, standard deviation, and critical variables of interest. Inferential statistics, including correlation coefficients (Pearson’s r) and multiple linear regression, addressed the other research questions. Standard multiple linear regression was chosen to evaluate the unique contribution of each predictor simultaneously, as this approach was deemed appropriate because there was no theoretical rationale to determine a specific entry order for the predictors.

## Results

### Sociodemographic characteristics of the study sample

Data were collected from 125 nurses who participated in the study. The mean age of the nurses was (M = 32, SD ± 7.0). The average years of experience was approximately (M = 8, SD ± 5.6), and the average years of experience in the current department was approximately (M = 5, SD ± 3.66). The highest percentage of the participants was males (*n* = 69, 55.2%), and the majority of them had a bachelor’s degree in nursing (*n* = 111, 88.8%). The participants worked in various hospital departments and units; the highest percentage was from the critical care units (*n* = 29, 23.2%), and (*n* = 86, 68.8%) of them worked 40 to 48 h per week. Finally, most participants (*n* = 84, 67.2%) earned a salary between 500 and 999 JD. Table 1 shows more details about the participants’ sociodemographic characteristics.

**Table 1:** Sociodemographic characteristics of study participants (*N* = 125)

**Characteristics**	**M (SD)**
Age	32 (7.03)
Year of experience	8 (5.60)
Experience in the current department	5 (3.66)
***Characteristics***	***n (%)***
**Gender**	
Male	69 (55.2%)
Female	56 (44.8%)
**Level of Education**	
BSc	111 (88.8%)
Master	11 (8.8%)
PhD	3 (2.4%)
**Working Department**	
Operation room	27 (21.6%)
ICU/CCU	29 (23.2%)
Emergency ward	18 (14.4%)
Surgical ward	18 (14.4%)
Medical ward	25 (20.0%)
Hemodialysis unit	8 (6.4%)
**Working Hours Per Week**	
Less than 40 h	27 (21.6%)
40 h to 48 h	86 (68.8%)
More than 48 h	12 (9.6%)
**Salary**	
Less than 500 JD	30 (24.0%)
500–999 JD	84 (67.2%)
Equal or more than 1000 JD	11 (8.8%)

### Nurses’ perceived level of transformational leadership

This section presents the participants’ responses to the research questions regarding the level of nurses’ perception toward TL among Jordanian nurses working at governmental hospitals; the mean and standard deviation were calculated for each variable, as shown in Table 2. The mean total score for the scale was (M = 36.80, SD ± 15.84), indicates a moderate perception of TL style. Specifically, nurses perceived transformational leadership at a moderate level across all dimensions, with mean scores of (M = 1.92, SD ± 0.81) for idealized influence, (M = 1.92, SD ± 0.87) for inspirational motivation, (M = 1.67, SD ± 0.88) for intellectual stimulation, and (M = 1.84, SD ± 0.85) for individualized consideration.

**Table 2:** Nurses’ perceived level of transformational leadership

#	Item	M (SD)
**Idealized Influence**		**1.92 (0.81)**
1.	My leader has confidence in a promising future for this organization.	1.81 (0.83)
2.	My leader wants to help when the organization is under challenging circumstances.	2.02 (0.96)
3.	My leader is a man who upholds organizational values.	2.10 (0.94)
4.	My leader has a clear vision and mission for this organization.	1.91 (0.90)
5.	My leader has a high commitment to advancing this organization.	1.87 (0.96)
6.	My leader is proud of the park employees.	1.83 (0.84)
**Inspirational Motivation**		**1.92 (0.87)**
7.	The leader can inspire me.	2.03 (1.04)
8.	The leader encouraged me to continue achievement.	1.77 (0.99)
9.	A leader is someone optimistic.	1.95 (0.97)
10.	Leaders can arouse employee morale when working.	2.16 (1.12)
11.	My leader is an example.	1.70 (1.01)
**Intellectual Stimulation**		**1.67 (0.88)**
12.	The leader encouraged me to think out of ability (out of the box).	1.61 (1.09)
13.	The leader supports me in taking it job risk.	1.60 (0.86)
14.	The leader encouraged me to be creative at work.	2.04 (1.04)
15.	Leadership behavior makes me comfortable at work.	1.94 (1.26)
16.	The leader encouraged me to learn new things.	1.70 (0.91)
**Individualized Consideration**		**1.84 (0.85)**
17.	The leader knows my abilities at work.	1.94 (1.06)
18.	The leader encouraged me to be at work.	1.83 (0.86)
19.	The leader gave an example of a job that I did not understand.	1.66 (0.97)
20.	The leader is someone who wants to listen to criticism/suggestions from employees.	1.93 (0.97)
**Average mean (out of 4)**		**1.84 (0.79)**
**Mean total score (out of 80)**		**36.8 (15.84)**

### Levels of nurses’ work motivation and engagement

#### Work motivation

The findings indicate that levels of work motivation among nurses working in Jordan’s government hospitals were moderate; the mean score for the scale was (M = 4.30, SD ± 1.40). The findings also showed that the levels of work motivation among nurses were moderate level across all subscales, with mean scores of (M = 4.38, SD ± 1.45) for intrinsic motivation, (M = 4.21, SD ± 1.50) for identified regulation of extrinsic motivation, (M = 4.43, SD ± 1.43) for introjected regulation of extrinsic motivation, and (M = 4.43, SD ± 1.43) for external regulation of extrinsic motivation. Further details are shown in Table 3.

**Table 3:** Levels of nurses’ work motivation

#	Item	M (SD)
1.	Because I enjoy this work very much.	4.43 (1.56)
2.	Because I have fun doing my job.	4.60 (1.56)
3.	For the moments of pleasure that this job brings me.	4.22 (1.57)
4.	I chose this job because it allows me to reach my life goals.	4.14 (1.72)
5.	Because this job fulfills my career plans.	3.96 (1.84)
6.	Because this job fits my values.	4.32 (1.69)
7.	Because I have to be the best in my job, I must be a “winner.”	4.61 (1.64)
8.	Because my work is my life, and I do not want to fail.	4.65 (1.59)
9.	Because my reputation depends on it.	4.20 (1.63)
10.	Because this job affords me a certain standard of living.	4.32 (1.40)
11.	Because it allows me to make a lot of money.	3.75 (1.70)
12.	I do this job for the paycheck.	4.37 (1.67)
**Average mean (out of 7)**		**4.30 (1.40)**
**Mean Total score (out of 84)**		**51.57 (16.75)**

#### Work engagement

The findings indicate that levels of work engagement among nurses working in Jordan’s government hospitals were moderate to high; the mean total score for the scale was (M = 80.5, SD ± 22.65). Regarding the subscales, the study findings showed that the levels of work engagement among nurses were at a moderate level across all domains, with mean scores of (M = 3.58, SD ± 1.37) for vigor domain, (M = 3.97, SD ± 1.46) for dedication domain, and (M = 3.69, SD ± 1.69) for absorption domain. Further details are shown in Table 4.

**Table 4:** Levels of nurses’ work engagement

#	Item	Mean (SD)
**Vigor Domain**		**3.58 (1.37)**
1.	At work, I feel like bursting with energy.	3.47 (1.53)
2.	At my job, I feel strong and vigorous.	3.47 (1.60)
3.	I feel like going to work when I get up in the morning.	2.86 (1.84)
4.	I can continue to work for long periods of time.	3.45 (1.50)
5.	At my job, I am mentally resilient.	4.16 (1.62)
6.	At my job, I always persevere, even when things do not go well.	4.10 (1.64)
**Dedication Domain**		**3.97 (1.46)**
7.	I find the work that I do meaningful and purposeful.	4.43 (1.58)
8.	I am enthusiastic about my job.	3.52 (1.69)
9.	My job inspires me.	3.64 (1.66)
10.	I am proud of the work that I do.	3.92 (1.67)
11.	My job is challenging enough.	4.35 (1.60)
**Absorption Domain**		**3.69 (1.42)**
12.	Time flies when I am at work.	4.04 (1.53)
13.	When I work, I forget everything else around me.	3.87 (1.51)
14.	I feel happy when I work intensively.	3.61 (1.79)
15.	I am immersed in my work.	3.66 (1.65)
16.	I get carried away when I work.	3.53 (1.68)
17.	It is difficult to detach myself from my job.	3.51 (1.73)
**Average mean (out of 6)**		**3.74 (1.33)**
**Mean Total score (out of 102)**		**80.50 (22.65)**

### The relationship between nurses’ perceived level of transformational leadership, work motivation, and work engagement

The results indicate that there is a statistically significant moderate positive correlation between the perceived level of TL and work motivation (*r* = .49, *p* < .001) as well as, there is a statistically significant moderate positive correlation (*r* = .41, *p* < .001) between the perceived level of TL and work engagement. Further, the results indicate a statistically significant positive correlation between work motivation and engagement (*r* = .85, *p* < .001).

### The predictors of work motivation and engagement

Multiple linear regression was performed to determine the predictors of work motivation and engagement among nurses. The first model was statistically significant F(7,112) = 6.56, *p* < .001, predicting 31.1% of the variance in nurses’ motivation. Similarly, the second model was statistically significant F(7,112) = 7.49, *p* < .001, and predicted 34.2% of the variance in nurses’ engagement. Furthermore, the tests indicated that TL style, years of experience, and weekly working hours were statistically significant predictors of nurses’ motivation and engagement. Notably, while transformational leadership had a positive association, both years of experience and weekly working hours demonstrated a negative relationship with motivation and engagement. Further details are shown in Table 5.

**Table 5:** Multiple linear regression analysis predicting nurses’ work motivation and engagement

Dependent variables	Variable	B	SE	β	t	*P*-value	95% Confidence Intervals for B	
							Lower	Upper
Motivation	(Constant)	53.684	8.423		6.373	< 0.001	37.25	68.56
	Transformational Leadership	0.452	0.099	0.342	4.550	**< 0.001**	0.17	0.58
	Working department	-1.165	1.338	-0.143	0.123	0.902	-3.70	0.52
	Years of experience	-6.429	1.747	-0.087	−3.679	**0.021**	-1.20	0.69
	Experience in the current department	1.748	1.258	0.148	1.389	0.168	-0.48	1.85
	Working hours/week	-5.501	2.697	-0.143	-2.040	**< 0.001**	-9.59	1.22
	Salary	-2.058	3.810	0.085	-0.540	0.590	-9.96	5.24
Engagement	(Constant)	98.991	11.141		8.886	-0.001	47.12	114.59
	Transformational Leadership	0.499	0.132	0.277	3.792	**< 0.001**	0.13	0.647
	Working department	-0.950	1.770	-0.307	-0.537	0.593	-7.19	-1.73
	Years of experience	-9.950	4.013	-0.129	-2.479	.**015**	-0.73	1.71
	Experience in the current department	0.619	0.737	0.035	0.841	0.403	-1.28	1.71
	Working hours/week	-10.274	3.566	-0.186	-2.881	**0.005**	-14.08	-0.12
	Salary	-3.257	5.038	-0.144	-0.646	0.519	-15.04	4.60

B = Unstandardized coefficient; SE = Standard error; β = Standardized coefficient;*p* < 0.05 indicates statistical significance

## Discussion

### Nurses’ perceived level of transformational leadership

The study results showed that Jordanian nurses working in governmental hospitals had a moderate level of perception toward TL. This study result is consistent with previous studies [36,37]. A possible explanation for this may be that the hierarchical organizational structures and traditional leadership approaches in Jordanian governmental hospitals may influence the behaviors of leaders. Cultural expectations surrounding authority and decision-making may also play a role, where leadership is viewed more as directive than collaborative. Leaders may find it difficult to implement TL approaches without autonomy, resistance to change, and communication gaps. Moreover, leadership training may also play an important role in shaping the effectiveness of transformational leadership. The ability of leaders to use this style may be affected by insufficient training opportunities that focus on strengthening the leadership capacities needed for TL. Further, nurses’ past experience with former leaders might affect how they perceive the current leadership efforts [28,38,39,40].

### Levels of nurses’ work motivation and engagement

The study results showed that Jordanian nurses working in governmental hospitals had moderate work motivation. These findings are consistent with other previous studies [41,42]. However, it must be noted that other studies indicated varying levels of work motivation among nurses (12,43–44), considering the differences in tools used. Factors such as work environment stressors, workload, limited resources, job security, limited opportunities for professional development, and low levels of recognition or reward may explain the moderate motivation levels. This highlights the multifaceted nature of motivation, suggesting that addressing environmental and organizational factors is essential to improving motivation among nurses. Therefore, understanding these factors can help develop plans to boost nurses’ motivation, such as offering organizational and leadership support, improving the working environment, and providing more opportunities for professional development.

The findings showed moderate to high levels of work engagement among nurses working in Jordan’s government hospitals. This result is consistent with another previous study [45]. Nevertheless, it must be noted that other studies indicated varying levels of work engagement among nurses [46–48], considering the differences in tools used. Furthermore, a possible explanation for this result could be due to several factors, such as the supportive working environment fostered by the hospitals and leaders, a sense of collaboration among staff, job stability, and opportunities for professional growth. Such factors may create a more resilient workforce capable of sustaining engagement despite challenges.

### Relationship between nurses’ perceived level of transformational leadership, work motivation, and work engagement

The results indicated that there is a statistically significant moderate positive correlation between TL and both work motivation as well as work engagement. This result is consistent with previous studies’ findings, which revealed that effective TL can be adapted to create a positive environment for nurses, contributing to their motivation and performance (49–50). Moreover, the current study’s results are associated with Monje-Amor and Vázquez’s [51] study, indicating that transformational leaders promote work engagement by creating a supportive working environment, facilitating staff communications, providing professional development opportunities, and ensuring the availability of sufficient resources. These mechanisms explain how TL influences motivation and engagement, emphasizing the role of leader behaviors in shaping workplace dynamics and employee experiences. In other words, adopting TL strategies cultivates a supportive and productive working atmosphere, which fosters optimal staff performance and a positive work attitude, leading to higher motivation levels. Additionally, this strategy provides individualized consideration and intellectual stimulation, which enhance employee comfort, sense of excitement, and cognitive investment when performing tasks, leading to higher levels of engagement [52].

### Predictors of nurses’ work motivation and engagement

The results showed that TL predicted nurses’ motivation and engagement variance. This underscores the crucial role of healthcare organizations in promoting TL, as it encourages nurses to carry out their tasks efficiently and fosters innovation in healthcare services. This result is consistent with other studies highlighting the value of applying TL in healthcare settings, creating a motivating and engaging work atmosphere [53,54]. In addition, TL can build a supportive work environment that reduces stress and burnout, enhances job satisfaction, and lowers employee turnover, keeping nurses feeling valued and increasing staff retention. Furthermore, TL supports Jordan’s national health strategic goals by promoting effective teamwork and improving the quality of health services provided to citizens.

It is worth noting that both years of experience and weekly working hours were significant predictors of nurses’ motivation and engagement levels. The negative association between years of experience and these outcomes may reflect professional fatigue, diminished enthusiasm, and limited opportunities for advancement as nurses progress in their careers. This finding aligns with previous studies [55], which suggest that early-career nurses often demonstrate greater motivation and engagement, driven by a desire to apply newly acquired skills, establish their professional identity, and pursue opportunities for advancement. From a theoretical perspective, this also aligns with burnout theory [56], which explains that prolonged exposure to workplace stressors without sufficient recovery leads to emotional exhaustion, diminished motivation, and engagement, especially among more experienced nurses who may face role stagnation and repetitive job demands. Similarly, the negative relationship between weekly working hours and motivation and engagement may indicate that excessive workloads contribute to emotional exhaustion and decreased professional investment. Consistent with prior research (57–58), this underscores the role of manageable working hours in mitigating burnout and enhancing motivation. These insights point toward practical interventions such as workload management and career development planning to sustain nurse motivation and engagement. Collectively, these findings emphasize the importance of workload regulation and structured career development in maintaining high levels of motivation and engagement, particularly among experienced nursing professionals [57,59,60].

Finally, the lack of a significant association between salary and nurses’ motivation and engagement may be attributed to the complex interplay of intrinsic and extrinsic factors influencing these outcomes. While financial compensation is a recognized extrinsic motivator, recent studies suggest that intrinsic elements, such as supportive leadership, may play a more pivotal role in fostering motivation and engagement among nurses [61]. Similarly, the nonsignificant effect of working department may indicate that factors such as organizational culture and leadership practices exert a more consistent influence across departments, thereby minimizing the department’s role in shaping motivation and engagement [62]. This suggests that healthcare organizations should prioritize creating an environment that nurtures these intrinsic factors to enhance nurse motivation and engagement.

### Implications

This section highlighted the study’s findings’ practical implications for nursing practice, management, education, and healthcare policies.

#### Implications for nursing practice

The TL style enhances employee motivation and engagement by emphasizing nurses’ professional growth and development, promoting a supportive work environment, and supporting high-quality healthcare. This leadership style emphasizes individualized consideration and empowerment, enabling nurses to become more capable of making their own decisions and adapting to changes in the workplace, which increases their motivation and willingness to provide distinguished care for patients and improve the quality of healthcare provided. Moreover, TL promotes innovation in nursing practices and adopts new standards to improve positive interactions within the medical team and enhance teamwork among coworkers.

#### Implications for nursing management

Transformational leadership in nursing management enhances nurses’ motivation and engagement by building a supportive and motivating work environment. This leadership style promotes innovation by encouraging open communication, empowering nurses to suggest improvements, and supporting creative problem-solving within the team. This approach leads to improved organizational productivity, enhanced quality of care, and a strengthened dedication among nurses to their professional roles and responsibilities. In general, transformational leaders focus on meeting the personal needs of nurses and appreciating their efforts at work, which increases their sense of satisfaction and commitment. In addition, transformational leaders encourage nurses to adapt to changes and achieve superior performance even under changing circumstances. This contributes to reducing stress and burnout, thereby increasing staff retention.

#### Implications for nursing education

In nursing education, TL focuses on enhancing students’ desire for excellence and promotes the love of learning, thus fostering a sustainable learning culture. Incorporating TL principles into nursing curricula can motivate students to achieve outstanding academic and professional performance through a supportive and engaging learning environment, enhancing their commitment to the education process and encouraging interaction with training programs and curricula.

Educators who adopt this style emphasize the development of adaptive skills and strengthen students’ ability to provide outstanding health care to meet the requirements of their profession. This approach promotes innovation by encouraging critical thinking, valuing students’ input, and supporting creative problem-solving within the curriculum, which in turn enhances innovation, improves teaching methods, and increases students’ engagement in the educational process. Furthermore, the application of TL in nursing education enhances academic motivation and engagement in work by creating a culture of adaptability that helps students navigate the evolving healthcare landscape, reduce tension, and build resilience, which prepares them to deliver exceptional patient care and assume leadership roles in their future careers.

### Recommendations

Applying TL in the health sector requires the adoption of a set of recommendations, including the following:

1. Developing educational strategies, including advanced practical training, to equip nursing leaders with both TL skills and practical capabilities.
2. Creating opportunities for nurses to contribute ideas and participate in the decision-making process. This initiative promotes workplace innovation, increases staff’s sense of responsibility, motivates them, and encourages active engagement.
3. Providing nurses with the opportunity to assume responsibility and leadership roles, along with the necessary training they need to adapt to changes in the workplace and promote the concept of collaborative leadership.
4. Developing hospital policies that encourage team-building activities to enhance collaboration, communication, and trust among staff members.
5. Establishing clear recognition and reward systems to acknowledge nurses’ efforts and achievements fosters a culture of appreciation.
6. Providing nurses with the opportunities and resources required for continuous education and professional growth, ensuring they can advance their skills and knowledge in healthcare.
7. Establishing a continuous monitoring system to evaluate leadership style effectiveness and staff adaptability.

### Strengths and limitations

The study exhibits theoretical and methodological strengths. Theoretically, this study is one of the limited studies examining the relationship between TL, motivation, and engagement among Jordanian nurses. Methodologically, a descriptive, cross-sectional, and correlational methodology was utilized in this study, which successfully attained the desired sample size by using appropriate sampling techniques. This ensured that the statistical tests had sufficient power and were conducted within the anticipated margins of error and confidence intervals.

Regarding the study limitations, the use of a correlational research design limits the ability to establish causal relationships between the studied variables. Additionally, the employment of convenience sampling may compromise the representativeness of the sample, thereby restricting the generalizability of the findings to the broader nursing population. In addition, using a self-reported questionnaire could also lead to response biases, such as respondents providing socially desirable answers.

## Conclusions

According to the study’s findings, nurses working in governmental hospitals in Jordan perceive transformational leadership at a moderate level. Their levels of work motivation were also moderate, while their work engagement ranged from moderate to high. Furthermore, the results revealed a moderate positive association between transformational leadership style and both nurses’ motivation and engagement. Finally, the findings indicated that transformational leadership style, years of experience, and weekly working hours significantly predicted nurses’ work motivation and engagement.

## Data Availability

All data generated or analyzed during this study are included in this published article.

## References

[1] Labrague LJ. Relationship between transformational leadership, adverse patient events, and nurse-assessed quality of care in emergency units: the mediating role of work satisfaction. Australasian Emerg Care. 2024;27(1):49–56. 10.1016/j.auec.2023.08.001 37598031

[2] Muenjohn N, Ishikawa J, Muenjohn P, Memon MA, Ting H. The effect of innovation and leadership on performance in China and Vietnam. Asia Pac Bus Rev. 2021;27(1):101–10.

[3] Robbins B, Davidhizar R. Transformational leadership in health care today. Health Care Manag. 2020;39(3):117–21. 10.1097/HCM.0000000000000296 32701607

[4] Orukwowu U, Ene-Peter J. Perception of nurses toward nursing leadership as an influential factor to better healthcare outcome. IPS J Public Health. 2022;1(1):1–15.

[5] McCay R, Lyles AA, Larkey L. Nurse leadership style, nurse satisfaction, and patient satisfaction: a systematic review. J Nurs Care Qual. 2018;33(4):361–7. 29266044 10.1097/NCQ.0000000000000317

[6] Krepia V, Katsaragakis S, Kaitelidou D, Prezerakos P. Transformational leadership and its evolution in nursing. Progress Health Sci. 2018;8:185–90.

[7] Collins E, Owen P, Digan J, Dunn F. Applying transformational leadership in nursing practice. Nurs Stand. 2020;35(5):59–66. 10.7748/ns.2019.e11408 31840443

[8] ANCC. Magnet Model 2019 [Available from:https://www.nursingworld.org/organizational-programs/magnet/magnet-model/

[9] Aini Q, Dzakiyullah NR. Leadership styles in healthcare settings for hospital management and employee engagement. J Angiotherapy. 2024;8(5):1–7.

[10] Moon SE, Van Dam PJ, Kitsos A, editors. Measuring transformational leadership in Establishing nursing care excellence. Healthcare: MDPI; 2019. 10.3390/healthcare7040132 PMC6956304 31689901

[11] Carthon JMB, Hatfield L, Plover C, Dierkes A, Davis L, Hedgeland T, et al. Association of nurse engagement and nurse staffing on patient safety. J Nurs Care Qual. 2019;34(1):40–6. 29889724 10.1097/NCQ.0000000000000334 PMC6263830

[12] Baljoon RA, Banjar HE, Banakhar MA. Nurses’ work motivation and the factors affecting it: A scoping review. Int J Nurs Clin Practices. 2018;5(1):277.

[13] Kohnen D, De Witte H, Schaufeli WB, Dello S, Bruyneel L, Sermeus W. Engaging leadership and nurse well-being: the role of the work environment and work motivation—a cross-sectional study. Hum Resour Health. 2024;22(1):8. 38225620 10.1186/s12960-023-00886-6 PMC10788988

[14] Cao Y, Gao L, Fan L, Zhang Z, Liu X, Jiao M, et al. Effects of verbal violence on job satisfaction, work engagement and the mediating role of emotional exhaustion among healthcare workers: a cross-sectional survey conducted in Chinese tertiary public hospitals. BMJ Open. 2023;13(3):e065918. 10.1136/bmjopen-2022-065918 PMC10008349 36898752

[15] García-Moyano L, Altisent R, Pellicer-García B, Guerrero-Portillo S, Arrazola-Alberdi O, Delgado-Marroquín MT. A concept analysis of professional commitment in nursing. Nurs Ethics. 2019;26(3):778–97. 28812947 10.1177/0969733017720847

[16] Hisel ME. Measuring work engagement in a multigenerational nursing workforce. J Nurs Adm Manag. 2020;28(2):294–305. 10.1111/jonm.12921 31788903

[17] Davies T, Daniels I, Roelofse M, Dean C, Parker J, Hanlon C, et al. Impacts of COVID-19 on mental health service provision in the Western cape, South africa: the MASC study. PLoS ONE. 2023;18(8):e0290712. 37639441 10.1371/journal.pone.0290712 PMC10461815

[18] Huston C. Professional issues in nursing: Challenges and opportunities. 2021.

[19] Catton H. Global challenges in health and health care for nurses and midwives everywhere. Int Nurs Rev. 2020;67(1):4–6. 32083728 10.1111/inr.12578 PMC7165846

[20] Assi H, Rayan A, Eshah NF, Albashtawy M, Al-Ghabeesh SH, editors. Nurse managers’ authentic leadership and their relationship with work engagement among registered nurses. Nursing Forum; 2024.

[21] Atalla AD, Elseesy NA, El-Ashry AM, Mohamed SM, Felemban EM, Alharbi A, Gheith NA. Unveiling the hidden connection: investigating the relationship between shared leadership and missed nursing care. Int J Nurs Sci. 2025;12(1):12–8. 39990993 10.1016/j.ijnss.2024.12.012 PMC11846603

[22] Suliman M, Almansi S, Mrayyan M, ALBashtawy M, Aljezawi M. Effect of nurse managers’ leadership styles on predicted nurse turnover. Nurs Manag. 2023;30(5). 10.7748/nm.2020.e1956 32662259

[23] Alkarabsheh OHM, Jaaffar AH, Wei Fong P, Attallah Almaaitah DA, Mohammad Alkharabsheh ZH. The relationship between leadership style and turnover intention of nurses in the public hospitals of Jordan. Cogent Bus Manage. 2022;9(1):2064405.

[24] Abu Yahya O, Ismaile S, Allari RS, Hammoudi BM, editors. Correlates of nurses’ motivation and their demographic characteristics. Nursing forum. Wiley Online Library; 2019. 10.1111/nuf.12291 30508252

[25] Wei H, Sewell KA, Woody G, Rose MA. The state of the science of nurse work environments in the united states: A systematic review. Int J Nurs Sci. 2018;5(3):287–300. 31406839 10.1016/j.ijnss.2018.04.010 PMC6626229

[26] Lake ET, Sanders J, Duan R, Riman KA, Schoenauer KM, Chen Y. A meta-analysis of the associations between the nurse work environment in hospitals and 4 sets of outcomes. Med Care. 2019;57(5):353–61. 30908381 10.1097/MLR.0000000000001109 PMC6615025

[27] Cummings GG, Tate K, Lee S, Wong CA, Paananen T, Micaroni SP, et al. Leadership styles and outcome patterns for the nursing workforce and work environment: A systematic review. Int J Nurs Stud. 2018;85:19–60. 29807190 10.1016/j.ijnurstu.2018.04.016

[28] Cummings GG, Lee S, Tate K, Penconek T, Micaroni SP, Paananen T, et al. The essentials of nursing leadership: A systematic review of factors and educational interventions influencing nursing leadership. Int J Nurs Stud. 2021;115:103842. 33383271 10.1016/j.ijnurstu.2020.103842

[29] MOH. Ministry of health report. Amman Ministry of Health; 2022.

[30] Faul F, Erdfelder E, Buchner A, Lang AG. Statistical power analyses using G*Power 3.1: tests for correlation and regression analyses. Behav Res Methods. 2009;41(4):1149–60.10.3758/BRM.41.4.1149. 19897823 10.3758/BRM.41.4.1149

[31] Bass BM, Avolio BJ. Multifactor leadership questionnaire. Menlo Park. 2004.

[32] Gagné M, Forest J, Gilbert M-H, Aubé C, Morin E, Malorni A. The motivation at work scale: validation evidence in two languages. Educ Psychol Meas. 2010;70(4):628–46.

[33] Stover O. Job satisfaction, engagement, and motivation for nursing leadership among millennial registered nurses (Doctoral dissertation, Walden University).

[34] Schaufeli W, Bakker A, Salanova M. Utrecht work engagement Scale-9 (UWES-9)[Database record]. APA PsycTests. 2006.

[35] Torabinia M, Mahmoudi S, Dolatshahi M, Abyaz MR. Measuring engagement in nurses: the psychometric properties of the Persian version of Utrecht work engagement scale. Med J Islamic Repub Iran. 2017;31:15. 10.18869/mjiri.31.15 PMC5609325 28955665

[36] Jankelová N, Joniaková Z, editors. Communication skills and transformational leadership style of first-line nurse managers in relation to job satisfaction of nurses and moderators of this relationship. Healthcare: MDPI; 2021. 10.3390/healthcare9030346 PMC8003159 33803822

[37] Ferreira VB, Amestoy SC, Silva GTRd, Felzemburgh RDM, Santana N, Trindade LL, et al. Transformational leadership in nurses’ practice in a university hospital. Acta Paulista De Enfermagem. 2018;31:644–50.

[38] Kartika ND. The influence of transformational leadership on nurses’ performances in Indonesia. Am J Phys Educ Health Sci. 2024;2(1):11–6.

[39] Hussain M. The Effect of Transformational Leadership on Nurses’ Performance. 2016.

[40] Al-Shaikh HY, Al-Maamari MAA. The influence of transformational leadership behaviors on organisational innovation in Yemeni Hospitals–Conceptual analysis. Int J Intellect Hum Resource Manage (IJIHRM). 2020;1(02):18–24.

[41] Tegegne E, Deml YA, Yirdaw G, Bewket Y. Work motivation and factors associated with it among health professionals in Debre Markos comprehensive specialized hospital. Sci Rep. 2024;14(1):2381. 38286807 10.1038/s41598-024-52409-5 PMC10825199

[42] Ayalew F, Kibwana S, Shawula S, Misganaw E, Abosse Z, Van Roosmalen J, et al. Understanding job satisfaction and motivation among nurses in public health facilities of ethiopia: a cross-sectional study. BMC Nurs. 2019;18:1–13. 31636508 10.1186/s12912-019-0373-8 PMC6794848

[43] Myroshnychenko YO, Mamay A. Motivation management model and practical realization within the health care institutions. 2021.

[44] Purba K, Sudibjo K. The effects analysis of transformational leadership, work motivation and compensation on employee performance in PT. Sago nauli. Budapest international research and critics Institute (BIRCI-Journal). Humanit Social Sci. 2020;3(3):1606–17.

[45] Al-Hamdan Z, Bani Issa H. The role of organizational support and self‐efficacy on work engagement among registered nurses in jordan: A descriptive study. J Nurs Adm Manag. 2022;30(7):2154–64. 10.1111/jonm.13456 34415087

[46] Raji IA, Ladan S, Alam MM, Idris IT. Organisational commitment, work engagement and job performance: empirical study on nigeria’s public healthcare system. J Global Bus Advancement. 2021;14(1):115–37.

[47] Taqatqa MR, Aljbour RH. Jordanian faculty members: will autonomy increase work engagement? EuroMed. J Manag. 2017;2(2):164–81.

[48] Fiabane E, Giorgi I, Sguazzin C, Argentero P. Work engagement and occupational stress in nurses and other healthcare workers: the role of organisational and personal factors. J Clin Nurs. 2013;22(17–18):2614–24. 23551268 10.1111/jocn.12084

[49] Alluhaybi A, Usher K, Durkin J, Wilson A. Clinical nurse managers’ leadership styles and staff nurses’ work engagement in Saudi arabia: A cross-sectional study. PLoS ONE. 2024;19(3):e0296082. 38452098 10.1371/journal.pone.0296082 PMC10919612

[50] Smama’h Y, Eshah NF, Al-Oweidat IA, Rayan A, Nashwan AJ. The impact of leadership styles of nurse managers on nurses’ motivation and turnover intention among Jordanian nurses. J Healthc Leadersh. 2023:19–29. 10.2147/JHL.S394601 PMC9884098 36718174

[51] Monje-Amor A, Vázquez JPA, Faíña JA. Transformational leadership and work engagement: exploring the mediating role of structural empowerment. Eur Manag J. 2020;38(1):169–78.

[52] Wu W-L, Lee Y-C. Do work engagement and transformational leadership facilitate knowledge sharing? A perspective of conservation of resources theory. Int J Environ Res Public Health. 2020;17(7):2615. 32290352 10.3390/ijerph17072615 PMC7177304

[53] Kodama Y, Fukahori H, Sato K, Nishida T. Is nurse managers’ leadership style related to Japanese staff nurses’ affective commitment to their hospital? J Nurs Adm Manag. 2016;24(7):884–92. 10.1111/jonm.12392 27145000

[54] Choi SL, Goh CF, Adam MBH, Tan OK. Transformational leadership, empowerment, and job satisfaction: the mediating role of employee empowerment. Hum Resour Health. 2016;14:1–14. 27903294 10.1186/s12960-016-0171-2 PMC5131441

[55] Astuty I, Udin U. The effect of perceived organizational support and transformational leadership on affective commitment and employee performance. J Asian Finance Econ Bus. 2020;7(10):401–11.

[56] Maslach C, Schaufeli WB, Leiter MP. Job burnout. Annu Rev Psychol. 2001;52:397–422.10.1146/annurev.psych.52.1.397. 11148311 10.1146/annurev.psych.52.1.397

[57] Van Bogaert P, Peremans L, Van Heusden D, Verspuy M, Kureckova V, Van de Cruys Z, et al. Predictors of burnout, work engagement and nurse reported job outcomes and quality of care: a mixed method study. BMC Nurs. 2017;16:1–14. 28115912 10.1186/s12912-016-0200-4 PMC5241948

[58] Khan BP, Griffin MTQ, Fitzpatrick JJ. Staff nurses’ perceptions of their nurse managers’ transformational leadership behaviors and their own structural empowerment. JONA: J Nurs Adm. 2018;48(12):609–14. 10.1097/NNA.0000000000000690 30407928

[59] Boamah SA, Laschinger HKS, Wong C, Clarke S. Effect of transformational leadership on job satisfaction and patient safety outcomes. Nurs Outlook. 2018;66(2):180–9. 29174629 10.1016/j.outlook.2017.10.004

[60] Mudallal RH, Othman WM, Al Hassan NF. Nurses’ burnout: the influence of leader empowering behaviors, work conditions, and demographic traits. INQUIRY: J Health Care Organ Provis Financing. 2017;54:0046958017724944. 10.1177/0046958017724944 PMC5798741 28844166

[61] Holtan A, et al. The role of leadership in nurses’ wellbeing and performance: A cross-sectional survey using a dual motivational pathway model. J Adv Nurs. 2024.10.1111/jan.16084. 10.1111/jan.16084 38318886

[62] Mazzetti G, Schaufeli WB. The impact of engaging leadership on employee engagement and team effectiveness: A longitudinal, multi-level study on the mediating role of personal- and team resources. PLoS ONE. 2022;17(6):e0269433.10.1371/journal.pone.0269433. 35767536 10.1371/journal.pone.0269433 PMC9242457

